# Infectious endophthalmitis associated with umbilical infection in Japanese black calf: a case report

**DOI:** 10.3389/fvets.2025.1567426

**Published:** 2025-04-25

**Authors:** Reiichiro Sato, Atsushi Iguchi, Ryoko Uemura, Hiroki Tsujita, Adrian Steiner

**Affiliations:** ^1^Graduate School of Medicine and Veterinary Medicine, University of Miyazaki, Miyazaki, Japan; ^2^Graduate School of Agriculture and Engineering, University of Miyazaki, Miyazaki, Japan; ^3^Veterinary Ophthalmology Specialized Clinic, Osaka, Japan; ^4^Vetsuisse Faculty, Clinic for Ruminants, University of Bern, Bern, Switzerland

**Keywords:** endophthalmitis, keratitis, third eyelid flap procedure, umbilical artery, urachus, ultrasonography

## Abstract

A 3-day-old Japanese black calf presented with a swollen and tender umbilical cord and diffusely cloudy and keratoconus eyes. Abdominal ultrasonography confirmed mild enlargement of both umbilical arteries and the urachus with a hyperechoic lumen. Additionally, a hyperechogenic structure suggestive of pus was noted near the abdominal wall. Fluorescein staining revealed corneal epithelial injury, whereas slit lamp examination identified corneal edema, increased corneal thickness, and keratitis with vascularization of the corneal stroma. Based on these findings, diagnoses of omphaloarteritis, omphalourachitis, and bullous keratitis were made. Both umbilical arteries and the urachus were surgically removed; both ocular globes were covered with a third eyelid flap, which was released 30 days postoperatively. On the follow-up, ocular ultrasonography indicated bleeding or fibrin deposits in the vitreous body of the right ocular globe. Because intraocular inflammation was suspected, anterior aqueous humor was collected from the right ocular globe, and bacterial examination was performed with the umbilical artery abscess, urachal abscess, and intraabdominal pus collected intraoperatively. *Escherichia coli* was isolated from the umbilical artery abscess, urachal abscess, intraabdominal pus, and aqueous humor, and all isolates exhibited identical genotypes. These findings suggest that endophthalmitis occurred as a result of the hematogenous spread of bacteria originating from septic umbilical cord remnants and that ocular ultrasonography is useful for assessing intraocular pathologies.

## 1 Introduction

In calves, at birth, the intraabdominal umbilical structures comprise a single umbilical vein, paired umbilical arteries, and the urachus (1, 2). The umbilical vein supplies oxygenated, nutrient-rich blood to the fetus via the liver and venous duct (1, 3, 4). The paired umbilical arteries, which branch from the internal iliac arteries, transport waste materials and deoxygenated blood from the fetus to the placenta (1, 3–5). The urachus connects the fetal bladder to the allantoic sac (1, 4–6). Umbilical vein infection is the second most common type of umbilical cord infection, following urachus infection, and occurs in 1%–14% of newborns (7). Umbilical arteries are less susceptible to infection than the urachus and umbilical veins (8). Although there are reports of cases where the infection has spread to localized sites, such as to the joints via the umbilical artery (9), they are rare.

Herein, we report the case of a calf that developed endophthalmitis after the infection spread from its umbilical artery to the ocular globe. Endophthalmitis refers to the inflammation of the interior cavity of the ocular globe and is usually caused by an infection. It is classified as either exogenous, with the infectious agent penetrating the cornea or sclera, or endogenous, with the infectious agent spreading hematogenously (10, 11). Endogenous endophthalmitis has been previously reported in cattle, and in these cases, the prognosis is poor due to sepsis caused by *Escherichia coli (E. coli)* or *Citrobacter koseri* (12–14). In humans, endogenous endophthalmitis has poor prognosis when the infection is caused by gram-negative bacteria or filamentous fungi (15–17).

This case report describes and discusses the potential of umbilical infection to cause endophthalmitis in cattle and emphasizes the utility of ocular ultrasonography of the ocular globe when opacity prevents complete examination of the eye.

## 2 Case description and diagnostic assessment

**Figure 1:**
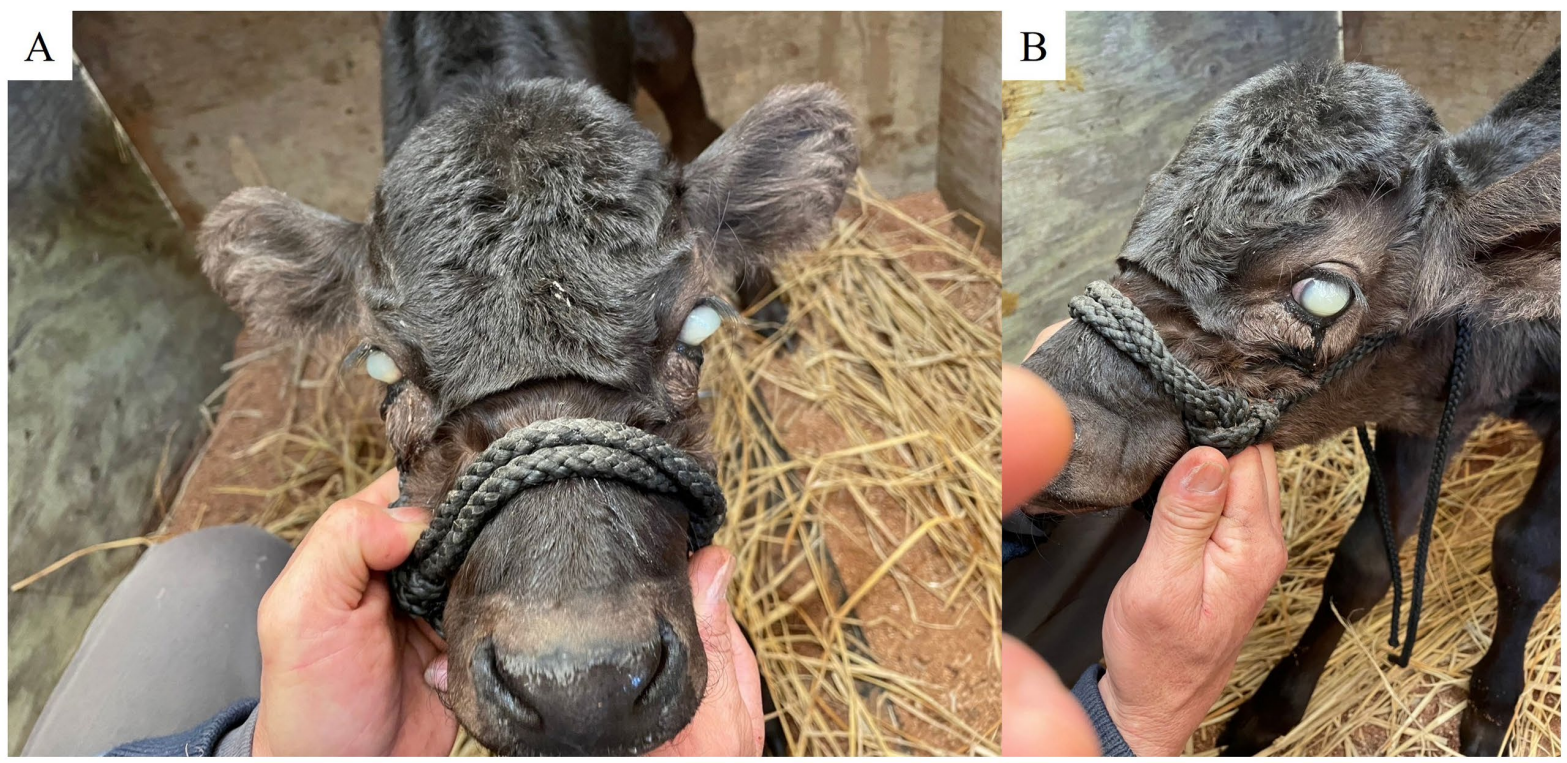
Corneal cloudiness, severe edema, and keratoconus in both eyes on Day 1. **(A)** Frontal view. **(B)** Left profile.

A 3-day-old female Japanese black calf presented with a fever (39.5°C), reduced appetite, and poor general condition. Physical examination by a local veterinarian revealed swelling and tenderness in the umbilical region, but there was no discharge of pus. Furthermore, cloudiness of cornea, severe edema, and keratoconus were observed in both eyes (Figure 1). On intravenous administration of cefazolin (1 mg/kg; Cefazolin-Chu; Fujita Pharmaceutical, Tokyo, Japan) by the local veterinarian once a day for 3 days, the calf’s rectal temperature temporarily decreased to 38.5°C. However, several days later, the fever recurred, and her eye condition worsened. Consequently, the calf was referred to the Miyazaki University Veterinary Teaching Hospital for further diagnostic and prognostic evaluation and potential treatment.

At the time of admission to the teaching hospital, the 18-day-old calf weighed 28 kg and demonstrated the following vital signs: heart rate, 60 beats/min; respiratory rate, 20 breaths/min; and rectal temperature, 39.8°C. Mild swelling, induration and tenderness were noted in the umbilical region. Furthermore, there was diffuse cloudiness and corneal bullae with keratoconus of both eyes evident. Although the calf appeared generally alert, it displayed signs of discomfort when the umbilical area was examined.

Complete blood count examination showed leukocytosis, particularly neutrophilia (white blood cells count, 23,000 cells/μL; neutrophil count, 19,000 cells/μL). Serum examination showed no abnormalities.

Abdominal ultrasonography (7.0–8.0-MHz variable linear probe, iViz air; FUJIFILM, Tokyo, Japan) confirmed the mild enlargement of both umbilical arteries and the urachus, with a hyperechogenic lumen. Additionally, a hyperechogenic image suggestive of pus and/or fibrin was observed near the abdominal wall. The presence of ascites was not observed.

**Figure 2:**
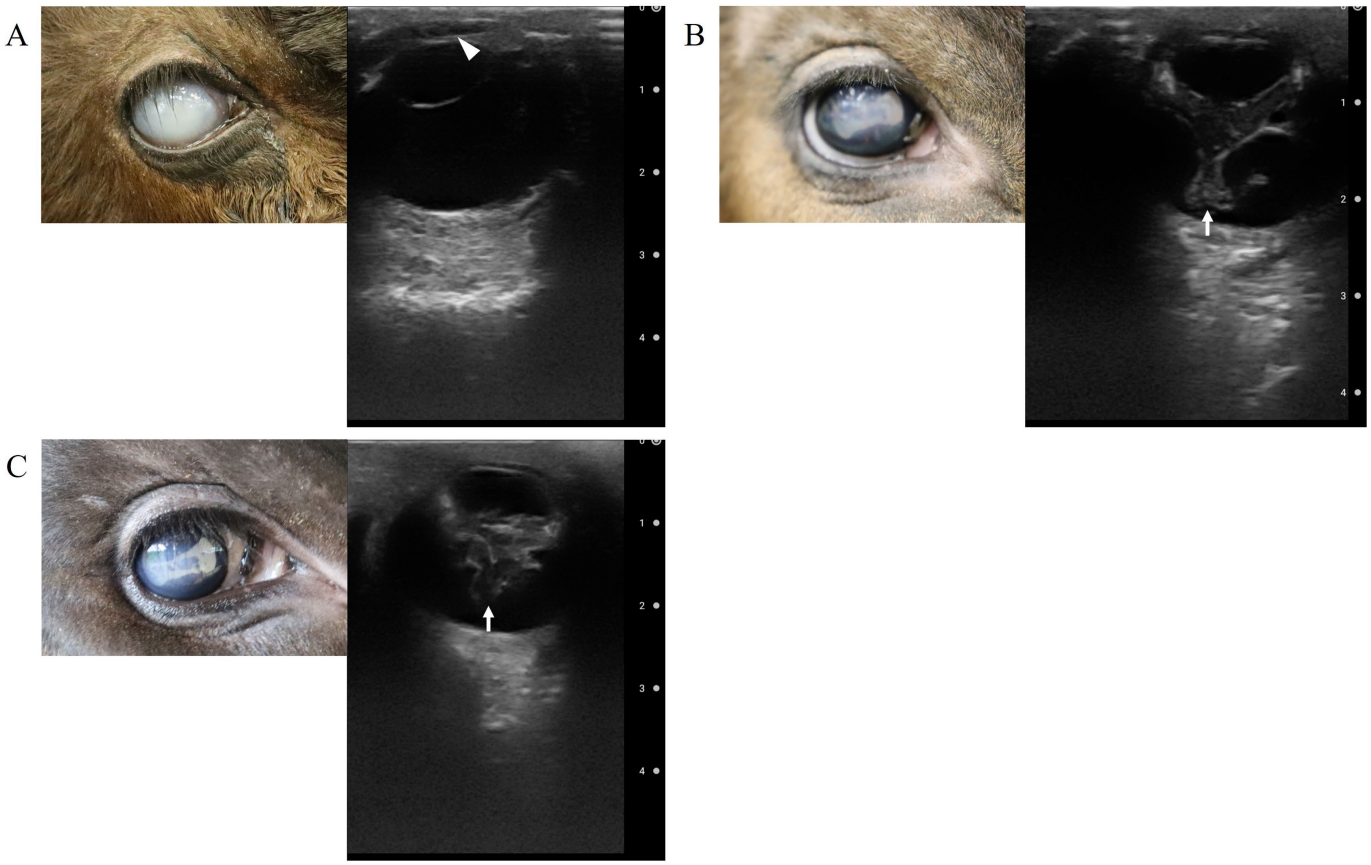
Ultrasound findings of the right eye: **(A)** at the time of the patient’s arrival. The opacity (fibrin/hypopyon) in the anterior chamber (arrow head), **(B)** 30 days after surgery (after the removal of the third eyelid flap), and **(C)** 40 days after surgery. Abnormalities in the vitreous body (arrows).

Fluorescein staining revealed epithelial injury of the cornea, slit-lamp examination (KOWA, SL-VVA150, Japan) identified corneal edema, increased corneal thickness, and keratitis with vascularization of the corneal stroma. An intraocular pressure test using a tonometer (TONOVET icare, Icare Finland, Vantaa, Finland) showed 13 mmHg in the left eye and 8 mmHg in the right eye (reference range, 7–25 mmHg) (18). Ocular ultrasonography (7.0–8.0-MHz variable linear probe, iViz air, linear probe) showed no abnormalities in the left eye, but a slight increase in the echogenicity was observed in the anterior chamber of the right eye, suggesting the presence of anterior uveitis and fibrin within the anterior chamber (Figure 2A). Additionally, the multiple ocular defect (MOD) gene test performed at the Livestock Improvement Association of Japan had normal results.

Based on these findings, the calf was diagnosed with bilateral umbilical artery abscess, urachal abscess, and bullous keratitis of both eyes and anterior uveitis of the right eye.

### 2.1 Ethics statement

Ethical review and approval were not required for this animal study as it pertains to a clinical case report. Written informed consent was obtained from the owners for participation of their animal in the study.

### 2.2 Surgical intervention

For the umbilical region, the umbilical artery and urachal abscesses were removed surgically. For the eyes, a third eyelid flap procedure was employed.

In detail, the calf was housed in a pen with ad libitum access to water. It was fasted for 12 h preoperatively. One hour preoperatively, a compound antibiotic containing 200,000 units of benzylpenicillin-procaine and 250 mg of dihydrostreptomycin sulfate (0.05 mL/kg; Mycillin Sol; Meiji Seika Pharma, Tokyo, Japan) was administered intramuscularly and prophylactically and flunixin meglumine (2 mg/kg; Forvet50; MSD, Tokyo, Japan) was administered intravenously for pain relief.

The calf was sedated with intravenous xylazine hydrochloride (0.2 mg/kg, Selactar, Elanco Japan, Tokyo, Japan), placed in dorsal recumbency, and anesthetized through continuous administration of isoflurane (Isoflu; Zoetis Japan, Tokyo, Japan) by endotracheal tube at a concentration of 2% in 100% oxygen. Local anesthesia with procaine hydrochloride (Adsan; Riken Vets Pharma, Saitama, Japan) was administered subcutaneously around the umbilical and left paramedian areas. For exploration of the abdominal cavity, an 8-cm left paramedian incision was made, starting approximately 1 cm caudal to the umbilicus at the left side and 1.5 cm from the midline.

**Figure 3:**
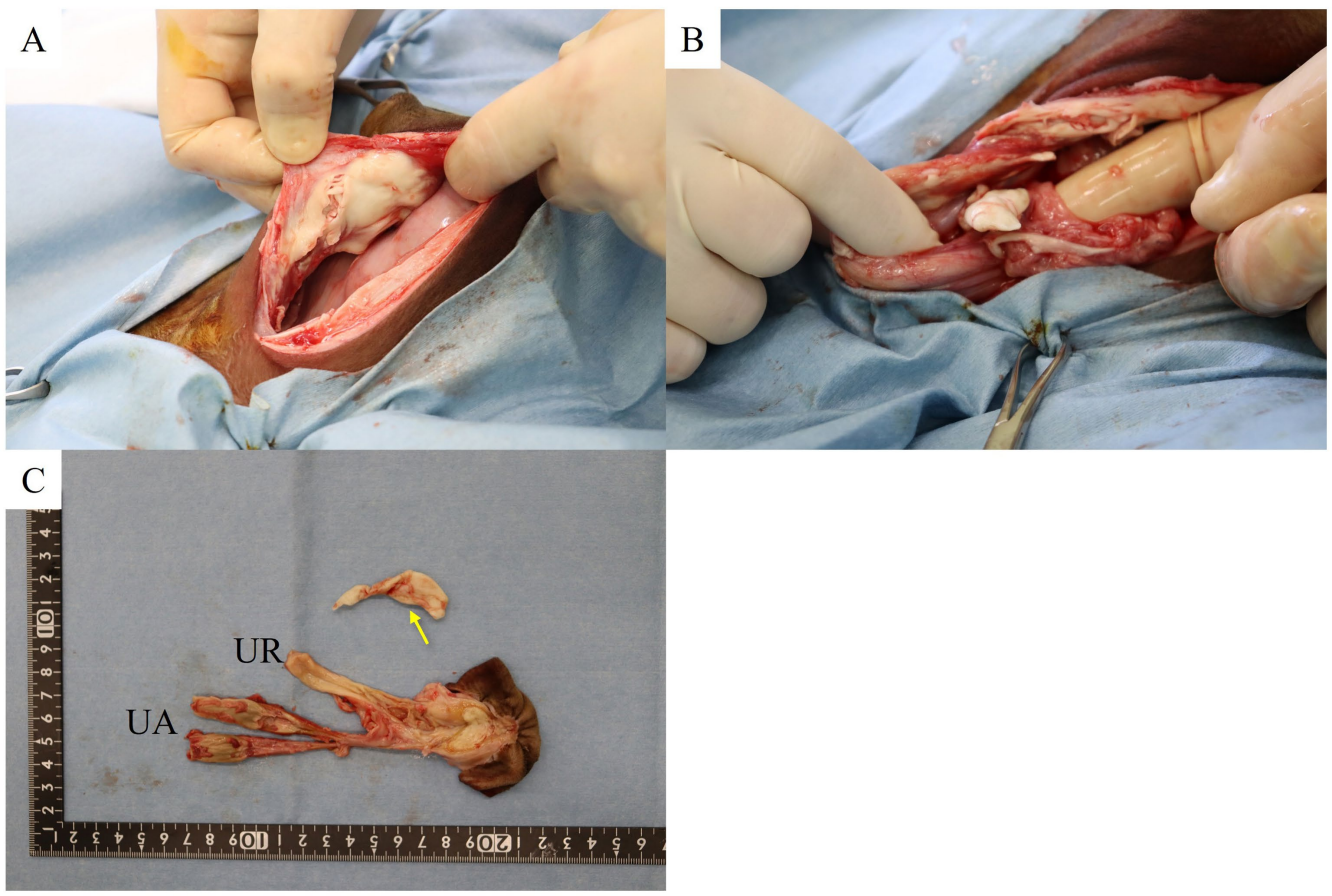
**(A)** Pyogenic granuloma adherent to the left parietal midline abdominal wall. **(B)** Pyogenic granuloma in the pelvic cavity. **(C)** Extracted remnant umbilical cord and pus mass in the abdominal cavity (arrow). UR, umbilical abscess; UA, umbilical artery abscess.

Abdominal-cavity exploration revealed a caseous clot containing pus and fibrin like material attached to the peritoneum near the umbilical cord (Figure 3A). Additionally, two more caseous clots likely containing pus and/or fibrin measuring approximately 2 cm in size were detected within the abdominal cavity (Figure 3B). The left and right umbilical arteries and urachus were mildly enlarged (Figure 3C). The umbilical arteries were ligated using a polyglycolic-acid synthetic absorbable suture material (Opepolyx, USP 3+4; Alfresa Pharma Corporation, Osaka, Japan) and sectioned. The urachus was mildly dilated from the base of the umbilicus to a point approximately 2 cm in caudal direction. Then, it narrowed and became fibrotic up to the pole of bladder. It was ligated approximately 4 cm from the bladder apex using a polyglycolic-acid synthetic absorbable suture material (Opepolyx, USP 3+4), and sectioned. The base of the umbilicus was hollowed out in a circle, and the umbilical artery and urachus were removed from the abdominal cavity along with the umbilicus. The umbilical vein was connected to the liver as a thin tube from the umbilicus, which we did not treat.

The peritoneum, internal and external rectus sheaths, and the abdominal rectus muscle were closed with a continuous suture pattern, using a polyglycolic-acid synthetic absorbable suture material (Opepolyx USP 3+4). The subcutaneous tissue was closed with a continuous suture pattern utilizing a polyglactin 910 synthetic absorbable suture material (coated VICRYL, USP 0; Ethicon, Bridgewater, NJ, United States). The skin was closed with an intradermally buried suture, using a synthetic absorbable thread (coated VICRYL USP 0).

Next, a third eyelid flap procedure was performed on both eyes. To prevent pressure necrosis of the eyelid at the suture site, an approximately 2-cm long feeding tube (4Fr, Atom Medical Corporation, Tokyo, Japan) was placed on the upper eyelid (at 1 cm from the edge of the eyelid). Non-absorbable suture material (ETHILON, USP 4–0; Johnson & Johnson, Tokyo, Japan) was used and passed from above the feeding tube, and the needle was passed from the conjunctival fornix of the upper eyelid. To prevent the nictitating membrane from tearing, the suture was passed through the free edge of the nictitating membrane in the same manner as a horizontal mattress suture, such that the thread was caught on the nictitating membrane cartilage. Once again, the needle was inserted through the conjunctival fornix of the upper eyelid at about 1 cm away from the first insertion point. The suture material was then passed through the lumen of the feeding tube, carefully tightened and knotted to completely cover the affected cornea.

Postoperative management included intramuscular administration of a compound antibiotic containing 200,000 units of benzylpenicillin-procaine and 250 mg of dihydrostreptomycin sulfate (0.05 mL/kg; Mycillin Sol; Meiji-Seika Pharma, Tokyo, Japan) for 5 days along with flunixin meglumine (2 mg/kg; Forvet50) intravenously the day after surgery for pain relief.

Additionally, eye drops, including 0.1% diclofenac sodium (Diclofenac Sodium Ophthalmic Solution 0.1%; ROHTONITTEN Co., Ltd. Aichi, Japan), 0.1% purified sodium hyaluronate (Hyalein ophthalmic solution 0.1%; Santen Pharmaceutical Co., Ltd. Osaka, Japan), and 1.5% levofloxacin hydrate (Cravit ophthalmic solution 1.5%; Santen Pharmaceutical Co., Ltd. Osaka, Japan), were administered thrice daily for 20 days after surgery.

### 2.3 Post-operative management

On the third postoperative day, the calf was returned to the farm. On the 30th postoperative day, both the third eyelid flap were removed. Edema and keratoconus in the left and right eyes had improved. Fluorescein staining was positive in a small area of both eyes, but the condition had improved significantly compared to the initial examination. The eye was assessed using ocular ultrasonography, and no abnormalities were found in the left ocular globe. In contrast, Y-shaped, membranous structures were present within the vitreous body of the right ocular globe that were heterogeneously echogenic and irregular in shape (Figure 2B).

As intraocular inflammation was suspected, 50 μL of the anterior aqueous humor was collected from the right ocular globe. The right eye was anesthetized with oxybuprocaine hydrochloride (Minims oxybuprocaine hydrochloride ophthalmic solution 0.4%, Senju Pharmaceutical Co., Ltd. Osaka, Japan) and rinsed with sterile saline. After draping and applying an eyelid retractor, the eye was stabilized with a cotton swab, and a 30G needle attached to a 1-cc aspirating syringe was inserted into the anterior chamber through the corneal limbus and aqueous humor. A bacterial culture of this sample was performed immediately. An additional course of a compound antibiotic containing 200,000 units of benzylpenicillin-procaine and 250 mg of dihydrostreptomycin sulfate (0.05 mL/kg; Mycillin Sol) was administered intramuscularly for 5 days, associated with 1.5% levofloxacin hydrate (Cravit ophthalmic solution 1.5%). A repeat ocular ultrasonography was performed 40 days postoperatively; however, the condition of the right eye remained unchanged (Figure 2C).

Additionally, the menace blink test results were positive for the left eye and negative for the right eye; results of the maze test to check for the presence or absence of vision strongly suggested that the presence of vision in the left eye and vision loss in the right eye.

At the time of sample collection from the umbilical artery and urachus abscesses, the intraabdominal pus/fibrin clots, and the anterior aqueous humor were collected and subsequently cultured on 5% sheep blood agar under both aerobic and anaerobic conditions at 37°C for 48 h. The cultured bacterial colonies were subjected to mass spectrometric analysis using a matrix-assisted laser desorption/ionization Biotyper (Bruker Daltonics Inc., Billerica, MA, United States), and *E. coli* was detected in all samples. Drug susceptibility testing was performed according to the protocol described by the Clinical and Laboratory Standards Institute (19). All *E. coli* strains were sensitive to cefazolin and enrofloxacin but resistant to penicillin and tetracycline.

Based on the results of the bacterial examination and susceptibility test, cefazolin sodium (5 mg/kg; Cefazolin-Chu; Fujita Pharmaceutical, Tokyo, Japan) was administered intravenously for 10 days.

Following the surgical intervention, the calf’s general condition gradually improved. At 21 months of age, it showed good development, similar to that of other cows of the same age living in the same stable. However, there was no recovery of vision in the right eye.

The drug susceptibility and phylogenetic relationships of four representative *E. coli* strains, SRE1 to SRE4, isolated from the umbilical artery abscess, urachal abscess, abdominal cavity fibrin clots, and anterior aqueous humor fluid, respectively, were investigated.

DNA-based *E. coli* O-serogroup (Og-type) were identified using 25 multiplex PCR kits (MP-1 to MP-25) targeting 162 Og-types corresponding to conventional O-serogroups (O1 O187) and 33 atypical Og-types (20, 21). Additionally, DNA-based *E. coli* H-type (Hg-type) were identified using 10 multiplex PCR kits (MP-A to MP-J) targeting 51 Hg-types from almost all conventional H-types (H1–H56) (22). None of the strains belonged to any Og-type (OgUT; untypeable), but their Hg-type was determined to be Hg25.

Phylogenetic analysis was also performed using multilocus sequence typing (MLST), which involved internal sequencing of seven housekeeping genes (*adk*, *fumC*, *gyrB*, *icd*, *mdh*, *purA*, and *recA*) (23). The sequence type (ST) was determined according to the EnteroBase MLST database.^1^ A phylogenetic tree was constructed based on the concatenated sequences (3,423 bp) of the seven genes used for MLST, employing the neighbor-joining method with the Tamura-Nei model in MEGA11 (24).

**Figure 4:**
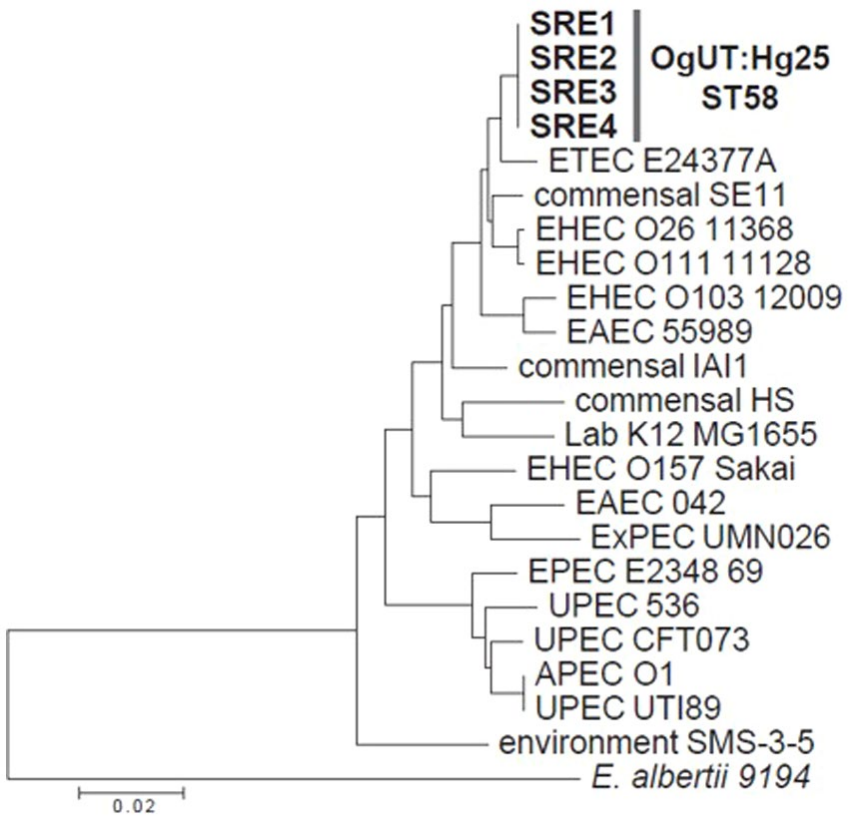
A phylogenetic tree of the four strains isolated in this study, along with 18 representative *Escherichia coli* strains and an *Escherichia albertii* strain, based on the concatenated sequences (3,423 bp) of seven housekeeping genes used for multilocus sequence typing.

All four *E. coli* strains were classified as ST58, belonging to *E. coli* phylogroup B1 (Figure 4).

## 3 Discussion

The umbilical vein supplies the fetus with oxygen- and nutrient-rich blood from the dam through from the dam through the umibilical cord and the liver (1, 3, 4). If the infection ascends, it may lead to liver abscess (2, 4). Furthermore, if bacteria spread hematogenously, they can cause arthritis and pneumonia, leading to poor prognosis and even death. Ompalophlebitis can also lead to significant economic losses (2, 4, 25, 26).

The umbilical artery, a branch of the internal iliac artery, carries waste products and deoxygenated blood from the fetus to the placenta, flowing in the opposite direction of the umbilical vein (1, 3–5). This reversed flow and the active retraction into the abdominal cavity in the course of umbilical involution makes the umbilical arteries less susceptible to infection compared with the umbilical vein or the urachus (8). However, a case has been reported wherein the infection spread to localized areas, such as the joints, via the umbilical artery (9).

Endogenous endophthalmitis is usually infectious. In cattle, septicemia can result in unilateral or bilateral endophthalmitis (12), with reports of infections caused by *Citrobacter koseri* (13) and *E. coli* (14). In human medicine, common risk factors include intravenous drug abuse, diabetes, indwelling catheters, and immunosuppression. Urinary tract infections and soft tissue abscesses have also been identified as potential sources of hematogenous spread infection (27, 28). Endogenous endophthalmitis can result from infection with various organisms, with *Klebsiella pneumoniae* and methicillin-resistant *Staphylococcus aureus* being notable examples. Endophthalmitis can cause corneal edema due to corneal endothelial damage, which leads to blisters under the corneal epithelium with subsequent corneal erosions if the blisters pop. Severe alteration to the corneal lamella can lead to corneal fibrosis.

Due to the nature of the disease where bacteria spread through the bloodstream, the microbial culture success rate in endogenous endophthalmitis is relatively low (28.6%) (29), underscoring the importance of early diagnosis and treatment (27–29). Therefore, administering broad-spectrum antibacterial and antifungal drugs early is crucial, even before the results of the culture are known, and treatment should be initiated even if the culture is negative. Furthermore, at the initial examination, rather than just focusing on obvious abnormalities of the eyes, all organ systems should be carefully examined for signs of trauma or abnormalities in the umbilical cord because these may be gateways for bacterial invasion. Particularly, if there are signs of a systemic condition, such as fever, there is a possibility of sepsis, and bacterial testing of the affected area and blood culture is thought to be useful.

In the current case, *E. coli* was isolated from the umbilical artery abscess, urachal abscess, intraabdominal pus, and anterior aqueous humor. Considering that the *E. coli* strains isolated from different sites exhibited identical genotypes, OgUT:Hg25-ST58, they were presumed to originate from the same clonal strain. These results suggested that *E. coli* OgUT:Hg25-ST58 entered the body through the umbilical cord after birth, proliferated in the umbilical remnants, triggered abscess formation and subsequently spread to the eye via the bloodstream, causing endophthalmitis. Furthermore, despite removal of the umbilical infection, which was the primary source, and the long-term (20 days) local antibacterial course after the eyelid flap, bacteria were still present in the right eye 30 days after surgery. To our knowledge, this is the first report to document endophthalmitis caused by an umbilical infection in cattle. When encountering ocular disease in calves, it is necessary to pay attention to the umbilical region as well as the eyes.

In this case, the vision in the right eye did not recover, and ocular ultrasonography of the right ocular globe revealed an image similar to the seagull sign with a hyperechogenic image in the vitreous body. However, the typical finding of retinal detachment with the detached retina remains fixed to the posterior wall of the eye at the optic nerve head and at the ora ciliaris retinae was not observed, so it could not be confirmed that it was a detached retina.

Retinal detachment can be a secondary condition of infectious endophthalmitis, and when a complete ocular examination is not possible due to cloudiness or other reason, an ultrasound examination of the eye is very effective for evaluating and diagnosing the internal eye (27, 28, 30). In veterinary medicine, it is used to diagnose various eye diseases in dogs (31), cats (32), horses (33, 34), goats and sheep (35), camels (36), and cows (17). It would also be helpful, when the cornea is too opaque to evaluate inside the eye, to use an ultrasound probe with a higher frequency that allows for better visualization of the anterior chamber.

## Data Availability

The original contributions presented in the study are included in the article/supplementary material, further inquiries can be directed to the corresponding author.

## Footnotes

^1^ http://enterobase.warwick.ac.uk/species/ecoli/allele_st_search
